# Tissue-Specific Expression of Monocarboxylate Transporters during Fasting in Mice

**DOI:** 10.1371/journal.pone.0112118

**Published:** 2014-11-12

**Authors:** Alexandra Schutkowski, Nicole Wege, Gabriele I. Stangl, Bettina König

**Affiliations:** Institute of Agricultural and Nutritional Sciences, Martin Luther University Halle-Wittenberg, Halle (Saale), Germany; University Claude Bernard Lyon 1, France

## Abstract

Monocarboxylates such as pyruvate, lactate and ketone bodies are crucial for energy supply of all tissues, especially during energy restriction. The transport of monocarboxylates across the plasma membrane of cells is mediated by monocarboxylate transporters (MCTs). Out of 14 known mammalian MCTs, six isoforms have been functionally characterized to transport monocarboxylates and short chain fatty acids (MCT1-4), thyroid hormones (MCT8, -10) and aromatic amino acids (MCT10). Knowledge on the regulation of the different MCT isoforms is rare. In an attempt to get more insights in regulation of MCT expression upon energy deprivation, we carried out a comprehensive analysis of tissue specific expression of five MCT isoforms upon 48 h of fasting in mice. Due to the crucial role of peroxisome proliferator-activated receptor (PPAR)-α as a central regulator of energy metabolism and as known regulator of MCT1 expression, we included both wildtype (WT) and PPARα knockout (KO) mice in our study. Liver, kidney, heart, small intestine, hypothalamus, pituitary gland and thyroid gland of the mice were analyzed. Here we show that the expression of all examined MCT isoforms was markedly altered by fasting compared to feeding. Expression of MCT1, MCT2 and MCT10 was either increased or decreased by fasting dependent on the analyzed tissue. MCT4 and MCT8 were down-regulated by fasting in all examined tissues. However, PPARα appeared to have a minor impact on MCT isoform regulation. Due to the fundamental role of MCTs in transport of energy providing metabolites and hormones involved in the regulation of energy homeostasis, we assumed that the observed fasting-induced adaptations of MCT expression seem to ensure an adequate energy supply of tissues during the fasting state. Since, MCT isoforms 1–4 are also necessary for the cellular uptake of drugs, the fasting-induced modifications of MCT expression have to be considered in future clinical care algorithms.

## Introduction

Monocarboxylates such as pyruvate, lactate and ketone bodies are central players in the metabolism of carbohydrates, lipids and amino acids and crucial for energy supply of all tissues, especially during food shortage. The transport of monocarboxylates across the plasma membrane of cells is mediated by monocarboxylate transporters (MCTs). Currently, 14 members of the MCT family, referred to as solute carrier family (SLC) 16, are described in mammals. Among them, six members have been functionally characterized so far (extensively reviewed in [1], [2]). The MCT isoforms 1–4 are proton-coupled transporters of monocarboxylates and also short-chain fatty acids [3]–[6]. For plasma membrane expression and activity, they require CD147 and embigin, respectively [7], [8]. MCT isoforms 1–4 vary in tissue and subcellular distribution and differ in their substrate specificity [1]. This allows shuttling of the substrates from tissues where they are produced to tissues that use them for oxidation or gluconeogenesis. MCTs are involved in shuttling lactate between skeletal muscle fibers [9], astrocytes and neurons [10], [11] and between tumor cells [12]. Attempts to target MCTs for tumor therapy [13] or immunosuppression [14] underline their important role also in pathophysiological processes. Moreover, MCT isoforms 1–4 are involved in the transport of several drugs such as salicylic acid, statins, γ-hydroxybutyrate and bumetanide [15], [16]. MCT8 is a specific thyroid hormone transporter [17]. Mutations in the MCT8 gene lead to severe psychomotor retardation [18] confirming the importance of MCT8 in thyroid hormone transport. MCT10, also referred to as TAT1, transports aromatic amino acid residues [19] and was recently found to transport also thyroid hormones [20], [21]. Even though data reveal an important impact of MCTs for both physiological and pathophysiological conditions, their regulation is not sufficiently described. Studies indicate that isoforms MCT1-4 are regulated at both transcriptional and post-transcriptional level [2]. Their expression can be modulated by substances like e.g. noradrenaline, insulin, IGF-1, butyrate or by other regulatory factors like exercise, hypoxia or the diabetic state. These modulations were shown to be linked to regulatory proteins like NF-κB, calcineurin, AMPK, PGC1α, HIF-1α and mTOR [22]–[28]. The regulation of the thyroid hormone transporters MCT8 and MCT10 remains virtually unknown [29]. Recently, we could demonstrate that MCT1 mRNA is up-regulated by peroxisome proliferator-activated receptor (PPAR)-α in the liver of rats, mice and pigs and also in the rat hepatoma cell line Fao [30], [31]. Increase of MCT1 mRNA in liver, kidney and small intestine upon stimulation of mice with a synthetic PPARα agonist was abolished in mice lacking PPARα (PPARα knockout (KO) mice), although a functional PPAR response element (PPRE) could not be found in the 5′-flanking region of mouse MCT1 gene [30], [31]. PPARα is a lipid-activated nuclear receptor that acts as a nutritional state sensor in mammalian cells and mediates the adaptive response to fasting by inducing fatty acid oxidation and ketogenesis [32], [33].

Though data on modulation of MCT expression by different substrates in selected tissues or cell types exist, there is a big gap of knowledge on metabolic regulation of MCTs. However, MCTs play a pivotal role in the distribution and tissue availability of energy substrates and regulators. By their additional function as drug transporters, any changes in MCT expression could cause alterations of drug deposition and pharmacokinetics. Therefore, it is crucial to investigate the MCT expression in response to feeding and fasting.

We carried out a comprehensive analysis of tissue specific expression of five MCT isoforms as well as the ancillary proteins CD147 and embigin upon 48 h of fasting in mice. Due to the crucial role of PPARα as a central regulator of energy metabolism and as known regulator of MCT1 expression, we included both wildtype (WT) and PPARα KO mice in our study.

## Materials and Methods

### Animal Studies and Dietary Procedures

Seven months old male PPARα KO mice (129S4/SvJae-Pparα^tm1Gonz^/J) and corresponding WT (control) mice (129S1/SvImJ) were purchased from Jackson Laboratory (Bar Harbor, ME, USA). The mice were kept in Macrolon cages in a room maintained with controlled temperature (23±1°C), humidity (50–60%), and lighting (6.00 a.m to 6.00 p.m). 32 mice of each genotype with an average initial body weight of 29.0±2.3 g (WT mice) and 28.8±2.1 g (PPARα KO mice) (mean ± SD, n = 32) were randomly assigned to two groups of 16 mice each. One group (“fed”) of each genotype received the commercial diet for rodents (“altromin 1324”, Altromin GmbH, Lage, Germany) *ad libitum* for the next 48 h (fed WT mice, n = 16; fed PPARα KO mice, n = 16), whereas from the other group (“fasted”) the diet was removed and mice were fasted for the next 48 h (fasted WT mice, n = 16; fasted PPARα KO mice, n = 16). Water was available *ad libitum* from nipple drinkers during the experiment. The study was approved by the respective regional government agency of Saxony-Anhalt (“Landesverwaltungsamt”, approval number H1-4/T1-10). All efforts were made to minimize suffering. Mice were then killed by decapitation under light anaesthesia with diethyl ether.

### Sample collection

Blood was collected into EDTA polyethylene tubes (Sarstedt, Nümbrecht, Germany). Plasma was obtained by centrifugation of the blood (1,100×g, 10 min, 4°C) and stored at −80°C. The hypothalamus was dissected by taking the anterior commissure as a horizontal reference and the line between the posterior hypothalamus and the mammillary bodies as the caudal limit [34]. The pituitary gland was dissected from the sella turcica, and the thyroid gland was excised. The small intestine (from pylorus to ileocecal valve) was completely excised and washed several times with cold NaCl solution (0.9%). Intestinal mucosa was harvested by scraping the surface of the small intestine. All tissues were immediately snap-frozen in liquid nitrogen and stored at −80°C pending analysis.

### Analysis of triacylglycerols and non-esterified fatty acids (NEFA)

For determination of triacylglycerol concentration in livers of mice, total liver lipids were extracted with a mixture of n-hexane and isopropanol (3∶2, v/v) [35]. After drying an aliquot of the lipid extract, the lipids were dissolved in Triton-X100 and chloroform (1∶1, w/w) [36]. Concentrations of triacylglycerol in liver lipid extracts were determined using an enzymatic reagent kit (DiaSys Diagnostic Systems, Holzheim, Germany). For the measurement of circulating NEFA, plasma of two mice from the same group was pooled. The concentration of NEFA was analyzed using an enzymatic reagent kit (Wako Chemicals GmbH, Neuss, Germany).

### Thyroid hormone analysis

For measurement of free thyroxine (fT4), plasma of two mice from the same group was pooled. Plasma concentration of fT4 was determined by means of an ELISA kit for analysis of fT4 (#CSB-E05080m, Cusabio, Wuhan, China) according to the manufacturer's instructions.

### Analysis of 3-hydroxybutyrate

The concentration of 3-hydroxybutyrate in plasma was determined using a commercially available kit (Wako Chemicals GmbH) according to the manufacturer's protocol.

### RNA isolation and real-time RT-PCR

Total RNA was isolated from tissues using Trizol reagent (Life Technologies, Darmstadt, Germany) according to the manufacturer's protocol. Pituitary glands of two mice and thyroid glands of two mice of the same group were pooled prior to RNA isolation. Total RNA concentration and purity were estimated from the optical density at 260 and 280 nm, respectively. A total of 1.2 µg of total RNA was used for cDNA synthesis using RevertAid M-MuLV reverse transcriptase (Thermo Fisher Scientific Inc., Waltham, MA, USA). For the determination of relative mRNA concentrations, real-time detection PCR using the Rotorgene 6000 system (Corbett Research, Mortlake, Australia) and SYBR Green I (Sigma-Aldrich, Taufkirchen, Germany) was applied. An aliquot of cDNA template was amplified in a total volume of 20 µl using 200 µM dNTPs (Genecraft, Cologne, Germany), 1.5 mM MgCl_2_, 0.5 U GoTaq DNA polymerase (both from Promega, Mannheim, Germany) and 5.4 pmol of each primer. The PCR protocol provided an initial denaturation at 95°C for 3 min and 20–35 cycles of amplification comprising denaturation at 95°C for 25 s, annealing at primer-specific temperatures (57–62°C) for 30 s and elongation at 72°C for 25 s. Subsequently, melting curve analysis was performed from 50 to 99°C with continuous fluorescence measurement. For determination of the mRNA concentration a threshold cycle (C_t_) and the amplification efficiency were obtained from each amplification curve using the software Rotor-Gene 4.6 (Corbett Research) and calculation of the relative mRNA concentration was performed according to [37]. In each tissue, several housekeeping genes were analyzed and their expression stability between all groups was checked by means of C_t_ values. Out of the housekeeping genes measured, for each tissue two to three most stable housekeeping genes were used for normalization. Primer pairs for embigin (NM_010330.4) and hypoxanthine guanine phosphoribosyl transferase (HPRT; NM_013556.2) were purchased from Sigma-Aldrich (www.kicqstart-primers-sigmaaldrich.com). Characteristics of all other primers used for PCR (Eurofins Genomics, Ebersberg, Germany) are shown in Table 1.

**Table 1:** Primer sequences used in real-time RT-PCR.

Gene name (Gene synonym)	Accession number	Forward primer (5′–3′)	Reverse primer (5′–3′)
3-hydroxy-3-methylglutaryl-coenzyme A synthase 2 (HMGCS2)	NM_008256.4	GGTGTCCCGTCTAATGGAGA	ACACCCAGGATTCACAGAGG
Beta-2 microglobulin (B2M)	NM_009735.3	TTCTGGTGCTTGTCTCACTGA	CAGTATGTTCGGCTTCCCATTC
CD147	NM_009768.2	ACTGGGGAAGAAGAGGCAAT	AACCAACACCAGGACCTCAG
Cyclophilin A (CypA)	NM_008907.1	GTGGTCTTTGGGAAGGTGAA	TTACAGGACATTGCGAGCAG
Monocarboxylic acid transporter 1 (MCT1)	NM_009196.4	CATTGGTGTTATTGGAGGTC	GAAAGCCTGATTAAGTGGAG
Monocarboxylic acid transporter 2 (MCT2)	NM_011391.1	CACCACCTCCAGTCAGATCG	CTCCCACTATCACCACAGGC
Monocarboxylic acid transporter 4 (MCT4)	NM_001038654.1	TCAATCATGGTGCTGGGACT	TGTCAGGTCAGTGAAGCCAT
Monocarboxylic acid transporter 8 (MCT8)	NM_009197.2	TGCCCTTGGTTACTTCGTCC	GGGACACCCGCAAAGTAGAA
Monocarboxylic acid transporter 10 (MCT10)	XM_006512864.1	TGTTCGGCTGCCGGAGAACA	TGACCAGTGACGGCTGGTAG
Ribosomal protein large P0 (RPLP0)	NM_007475.5	GAAACTGCTGCCTCACATCCG	CTGGCACAGTGACCTCACACG

### Western blot

Western blotting was performed as described earlier [38]. Tissue samples were lysed using RIPA buffer (50 mM Tris/HCl (pH 7.5), 150 mM NaCl, 1% Triton-X100, 0.5% sodium deoxycholate, 0.1% SDS, 5 mM EDTA) and tissue lyser. Twenty µg of protein lysate were used for SDS-PAGE. Cyclophilin A was used for normalization of protein expression data because its expression was not affected by fasting or genotype. The following antibodies were used: anti-MCT1 (ab90582, Abcam, Cambridge, UK), anti-MCT2 (sc-166925, Santa Cruz Biotechnology, Santa Cruz, CA, USA), anti MCT-8 (20676-1-AP, Proteintech, Chicago, IL, USA), anti-Cyclophilin A (ab41684, Abcam). Primary antibodies were detected using HRP-conjugated secondary antibodies using ECL Prime western blotting detection reagent (GE Healthcare, Munich, Germany).

### Statistical analysis

Values are expressed as means ± SD. Data were analyzed by two-way ANOVA, including the factors genotype, fasting, and the interaction of these factors using the software SPSS 20 (IBM, Armonk, NY, USA). Values were analyzed for homoscedasticity by Leven's test. In case of homogeneity of variance means, the four groups were compared by Tukey's test, or in case of unequal variances (concerning variance heterogeneity, effect was considered significantly different at *p*<0.05) by Games-Howell. Differences of *p*<0.05 in post-hoc comparison were considered to be significant.

## Results

### Effect of fasting on body and plasma parameters

**Figure 1:**
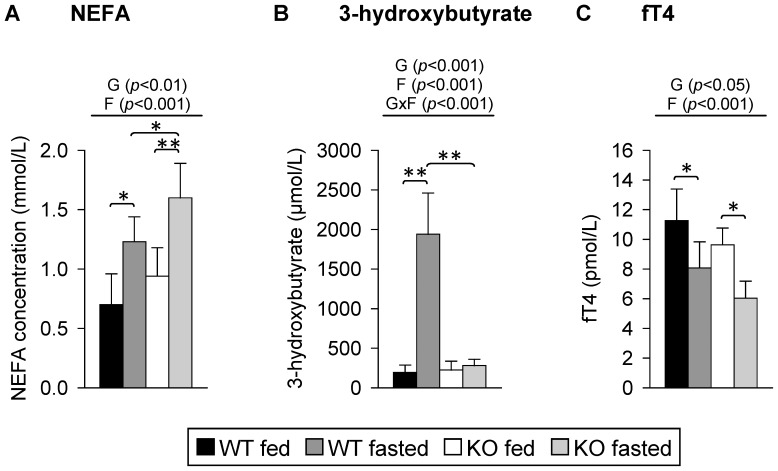
Final plasma concentrations of (A) non-esterified fatty acids (NEFA), (B) 3-hydroxybutyrate and (C) free thyroxine (fT4) in response to fasting and PPARα. Values represent means ± SD of plasma concentrations of wildtype (WT) and PPARα knockout (KO) mice that were fed *ad libitum* or fasted for 48 h (n = 16 for 3-hydroxybutyrate, n = 8 for NEFA and fT4). Data were analyzed by two-way ANOVA. Classification factors were genotype, fasting, and the interaction between both factors. *P*-values revealed by two-way ANOVA are noted above the figures. Individual means of the treatment groups were compared by Tukey's test in case of variance homogeneity. In case of variance heterogeneity, as revealed by Levene's test, individual means were compared by Games Howell test. Horizontal brackets represent differences between groups in post-hoc comparison (**p<0.001, *p<0.05).

Mice that were fasted for 48 h had lower final body weights than the fed mice, while the genotype had no effect on final body weight (WT mice fed, 28.6±3.1 g; WT mice fasted, 23.2±2.6 g; PPARα KO mice fed, 28.4±2.2 g; PPARα KO mice fasted, 23.4±3.0 g). Relative weights and triacylglycerol concentrations of livers of mice were influenced by both genotype (*p*<0.001) and fasting (*p*<0.01 and *p*<0.001, respectively) according to two-way ANOVA analysis. Fasted PPARα KO mice had higher relative liver weights than fed PPARα KO mice; those differences were not seen in the WT mice (*p*<0.001; WT mice fed, 4.07±0.45 g/100 g body weight; WT mice fasted, 3.77±0.31 g/100 g body weight; PPARα KO mice fed, 4.32±0.38 g/100 g body weight; PPARα KO mice fasted, 5.16±0.32 g/100 g body weight). Liver triacylglycerol concentrations were higher in fed and fasted PPARα KO mice compared to the corresponding groups of WT mice (*p*<0.001; WT mice fed, 9.4±2.0 µmol/g; WT mice fasted, 106.9±20.8 µmol/g; PPARα KO mice fed, 38.9±16.1 µmol/g; PPARα KO mice fasted, 230.4±44.7 µmol/g). Fasted WT and PPARα KO mice were characterized by higher plasma concentrations of NEFA than fed mice of the same genotype, whereby the highest NEFA concentrations were observed in fasted PPARα KO mice (Figure 1A). As expected, the circulating plasma concentration of 3-hydroxybutyrate upon fasting was increased in WT but not in the PPARα KO mice (Figure 1B). Plasma fT4 concentrations were lower in PPARα KO than in the WT mice. Fasted mice of both genotypes had lower plasma concentrations of fT4 than corresponding fed mice (Figure 1C).

### Fasting induced a typical PPARα target gene in mouse tissues

**Figure 2:**
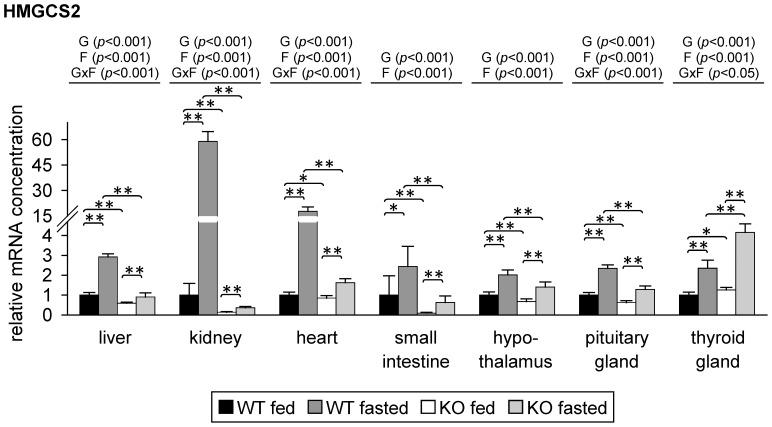
Relative mRNA concentrations of 3-hydroxy-3-methylglutaryl-coenzyme A synthase (HMGCS)-2 in mouse tissues in response to fasting and PPARα. Values represent means ± SD of relative mRNA concentrations of wildtype (WT) and PPARα knockout (KO) mice that were fed *ad libitum* or fasted for 48 h (n = 16 for liver, kidney, heart, small intestine and hypothalamus; n = 8 for pituitary gland, n = 6 for thyroid gland). Data were analyzed by two-way ANOVA. Classification factors were genotype, fasting, and the interaction between both factors. *P*-values revealed by two-way ANOVA are noted above the figures. Individual means of the treatment groups were compared by Tukey's test in case of variance homogeneity. In case of variance heterogeneity, as revealed by Levene's test, individual means were compared by Games Howell test. Horizontal brackets represent differences between groups in post-hoc comparison (**p<0.001, *p<0.05).

To demonstrate PPARα activation, we analyzed the relative mRNA concentration of a typical PPARα target gene, namely 3-hydroxy-3-methylglutaryl-coenzyme A synthase (HMGCS)-2 [39], in liver, kidney, heart, small intestine, hypothalamus, pituitary gland and thyroid gland of the mice. As expected, two-way ANOVA showed that the relative mRNA level of HMGCS2 was influenced by genotype in all tissues analyzed. In liver, kidney, heart, small intestine, hypothalamus and pituitary gland, relative mRNA concentrations of HMGCS2 were lower in PPARα KO mice than in corresponding WT mice (Figure 2). In these tissues, the relative mRNA concentration of HMGCS2 was higher in fasted than in fed WT mice (Figure 2). Strongest fasting-induced increase of HMGCS2 mRNA level in WT mice was observed in kidney and heart (about 60- and 17-fold, respectively); the increase in the other tissues was about 2-3-fold (Figure 2). The mRNA level of HMGCS2 was also induced upon fasting in these tissues of PPARα KO mice; in liver, kidney, heart and pituitary gland this increase was less pronounced than in fasted WT mice (Figure 2). Surprisingly, in thyroid gland, HMGCS2 mRNA concentration was higher in PPARα KO than in WT mice. Furthermore, the increase upon fasting was stronger in PPARα KO than in WT mice (about 4.2- and 2.4-fold, respectively) which is in contrast to the observations in the other tissues (Figure 2).

### Fasting differentially affected expression of MCT1, MCT2 and MCT4 in mouse tissues

**Figure 3:**
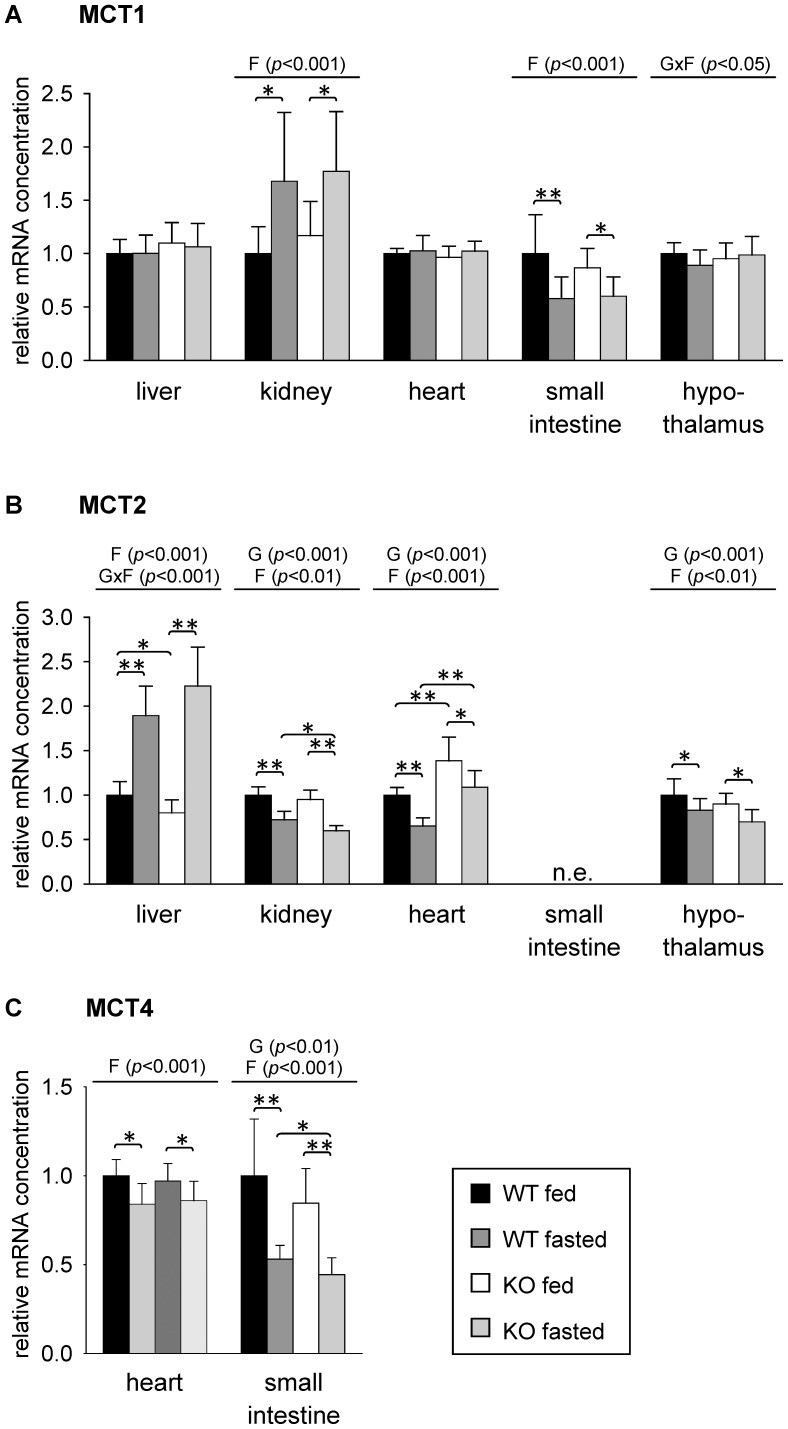
Relative mRNA concentrations of (A) monocarboxylate transporter (MCT)-1, (B) MCT2 and (C) MCT4 in mouse tissues in response to fasting and PPARα. Values represent means ± SD of relative mRNA concentrations of wildtype (WT) and PPARα knockout (KO) mice that were fed *ad libitum* or fasted for 48 h (n = 16). Data were analyzed by two-way ANOVA. Classification factors were genotype, fasting, and the interaction between both factors. *P*-values revealed by two-way ANOVA are noted above the figures. Individual means of the treatment groups were compared by Tukey's test in case of variance homogeneity. In case of variance heterogeneity, as revealed by Levene's test, individual means were compared by Games Howell test. Horizontal brackets represent differences between groups in post-hoc comparison (**p<0.001, *p<0.05). n.e., not evaluable due to extremely low expression.

The effect of 48 h fasting on expression of MCT1, MCT2 and MCT4 isoforms that preferentially transport ketone bodies, lactate and pyruvate was studied in liver, kidney, heart, small intestinal mucosa and hypothalamus. Using real-time detection RT-PCR, MCT1 mRNA could be detected in all of these tissues. As shown by two-way ANOVA, relative mRNA concentration of MCT1 was influenced by fasting in kidney and small intestine of the mice (Figure 3A). Relative MCT1 mRNA level was about 1.7-fold higher in kidneys of fasted mice compared to fed mice irrespective of genotype (Figure 3A). In small intestine, MCT1 mRNA concentration was about 40% lower in fasted than in fed mice of both genotypes (Figure 3A). No relevant changes of MCT1 mRNA concentration were observed in liver, heart and hypothalamus of mice (Figure 3A).

Relative concentration of MCT2 mRNA was influenced by fasting in liver, kidney, heart and hypothalamus and further by genotype in kidney, heart and hypothalamus. Liver MCT2 mRNA concentration was higher in fasted than in fed mice with a stronger fasting-induced increase in PPARα KO than in WT mice (2.8-fold and 1.9-fold, respectively, compared to fed mice of the respective genotype; Figure 3B). In contrast to liver, MCT2 mRNA was reduced upon fasting in kidney, heart and hypothalamus of mice of both genotypes. PPARα KO mice of both treatment groups had higher mRNA concentrations of MCT2 in hearts than the corresponding groups of WT mice (Figure 3B). In accordance with literature [39], MCT2 mRNA expression in small intestine could not be detected. In our study, expression of MCT4 mRNA could be detected in hearts of mice using real-time RT-PCR, although to a lesser extent than in small intestine (by comparison of cycle threshold values in real-time PCR, data not shown). No considerable expression was observed in the other tissues examined. In both, heart and small intestine, MCT4 mRNA concentration was influenced by fasting (Figure 3C). In small intestine, an additional influence of genotype was observed. Irrespective of the genotype, the relative MCT4 mRNA concentration was decreased upon 48 h of fasting by about 15% in heart and by about 50% in small intestine compared to feeding (Figure 3B).

**Figure 4:**
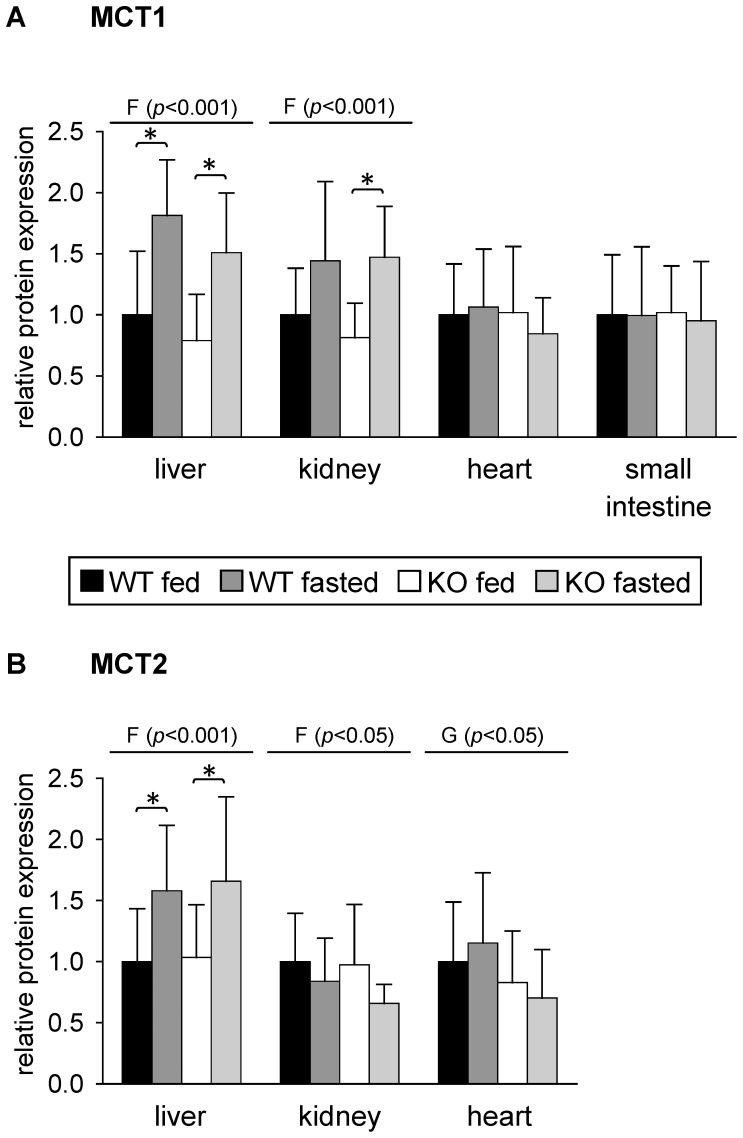
Relative protein expression of (A) monocarboxylate transporter (MCT)-1 and (B) MCT2 in mouse tissues in response to fasting and PPARα. Values represent means ± SD of relative protein expression of wildtype (WT) and PPARα knockout (KO) mice that were fed *ad libitum* or fasted for 48 h (n = 16). Data were analyzed by two-way ANOVA. Classification factors were genotype, fasting, and the interaction between both factors. *P*-values revealed by two-way ANOVA are noted above the figures. Individual means of the treatment groups were compared by Tukey's test in case of variance homogeneity. In case of variance heterogeneity, as revealed by Levene's test, individual means were compared by Games Howell test. Horizontal brackets represent differences between groups in post-hoc comparison (*p<0.05).

To analyze whether changes observed in relative mRNA concentration of MCTs resulted in alterations of protein expression, we performed western blot analysis of total tissue lysates using specific antibodies for MCT1 and MCT2 (Figure S1). Out of the tissues analyzed regarding mRNA concentration, we chose liver, kidney, heart and small intestine for protein expression analysis. As shown in Figure 4A, MCT1 protein expression in heart was not different between all four groups of mice analyzed thus confirming the results of mRNA analysis (Figure 3A). Protein expression of MCT1 was also not altered by treatment or genotype in small intestine of mice (Figure 4A). This is in contrast to the results of mRNA analysis where a reduction of MCT1 mRNA level upon fasting was observed in both genotypes (Figure 3A). Fasting increased MCT1 protein expression in livers of both WT and PPARα KO mice about 1.8- and 1.9-fold, respectively, compared to fed mice of the same genotype (Figure 4A). In contrast, no alterations in MCT1 mRNA concentration in liver were observed after 48 h of fasting in both genotypes (Figure 3A). MCT1 protein expression in kidney was also influenced by fasting. It increased about 1.9-fold in fasted compared to fed PPARα KO mice whereas in WT mice the increase upon fasting of about 1.4-fold narrowly failed significance level. Thus, protein expression data of MCT1 in kidney are in agreement with results of mRNA analysis (Figure 3A).

MCT2 protein expression was influenced by fasting in liver and kidney. In liver, fasted WT and PPARα KO mice had 1.6-fold higher MCT2 protein expression than the fed groups of mice (Figure 4B), thus confirming the results of MCT2 mRNA analysis in liver (Figure 2B). Fasting decreased MCT2 protein expression in kidneys of both WT and PPARα KO mice compared to respective fed mice about 16 and 32%, respectively, nevertheless due to large standard deviations these changes did not reach significance in post-hoc comparison (Figure 4B). However, a reduction of MCT2 protein expression in kidney upon fasting is in agreement with results of MCT2 mRNA analysis in kidney (Figure 3B). No significant differences in MCT2 protein expression between the treatment groups were found in heart. Thus, protein expression of MCT2 in heart does not match results of corresponding mRNA analysis (Figure 3B). Regarding MCT2 protein expression, small intestine was not analyzed since no reliable mRNA expression was found using real-time RT-PCR.

### Fasting also affected expression of MCT ancillary proteins in mouse tissues

MCT1, -2 and -4 require association with CD147 or embigin for proper membrane localization and functioning [1]. Thus, we also analyzed possible changes in relative mRNA concentrations of CD147 and embigin upon fasting of mice. Fasting decreased the relative mRNA concentration of CD147 in kidney, small intestine and hypothalamus of both WT and PPARα KO mice compared to fed mice (Figure S2). Decrease of kidney CD147 mRNA level upon fasting compared to the level of the corresponding fed group was more pronounced in PPARα KO than in WT mice (13% and 34%, respectively; Figure S2). In liver, a fasting-associated decrease of CD147 mRNA of about 20% was only observed in PPARα KO mice (Figure S2).

Using real-time RT-PCR, significant mRNA expression of embigin could only be detected in kidney and hypothalamus of mice. A slight but significant reduction of embigin mRNA concentration upon fasting compared to corresponding fed mice was observed in kidneys of PPARα KO mice but not of WT mice (Figure S2). In hypothalamus, embigin mRNA concentration was influenced by genotype with slight but significant lower expression in PPARα KO compared to WT mice (Figure S2).

### Fasting differentially affected expression of MCT8 and MCT10 in mouse tissues

**Figure 5:**
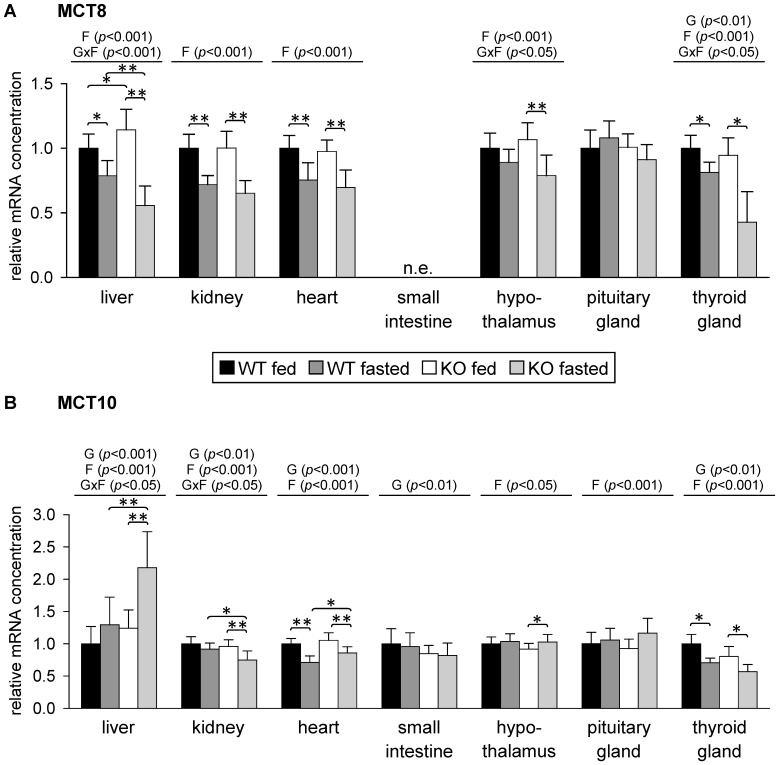
Relative mRNA concentration of (A) monocarboxylate transporter (MCT)-8 and (B) MCT10 in mouse tissues in response to fasting and PPARα. Values represent means ± SD of relative mRNA concentrations of wildtype (WT) and PPARα knockout (KO) mice that were fed *ad libitum* or fasted for 48 h (n = 16 for liver, kidney, heart, small intestine and hypothalamus, n = 8 for pituitary gland, n = 6 for thyroid gland). Data were analyzed by two-way ANOVA. Classification factors were genotype, fasting, and the interaction between both factors. *P*-values revealed by two-way ANOVA are noted above the figures. Individual means of the treatment groups were compared by Tukey's test in case of variance homogeneity. In case of variance heterogeneity, as revealed by Levene's test, individual means were compared by Games Howell test. Horizontal brackets represent differences between groups in post-hoc comparison (**p<0.001, *p<0.05). n.e., not evaluable due to extremely low expression.

We then studied the effect of 48 h fasting on expression of MCT8 and MCT10, isoforms that preferentially transport thyroid hormones and in case of MCT10 also aromatic amino acids. No reliable MCT8 expression was observed in small intestine of mice according to real-time RT-PCR analysis. MCT8 mRNA concentration was decreased by fasting in liver, kidney, heart and thyroid gland of both WT and PPARα KO mice (Figure 5A). In liver, MCT8 mRNA concentration was moderately higher in fed WT than in fed PPARα KO mice. Liver MCT8 mRNA concentration was lower in fasted than in fed mice with a stronger fasting-induced decrease in PPARα KO mice compared to WT mice (about 49% and 21%, respectively, compared to fed mice of the respective genotype; Figure 5A). In kidney and heart, relative mRNA concentration of MCT8 was about 25 to 35% lower in fasted than in fed mice with no significant differences between mice of different genotypes (Figure 5A). Fasting decreased hypothalamic MCT8 mRNA level about 26% in PPARα KO mice whereas in WT mice no significant reduction was observed (Figure 5A). In thyroid gland, relative mRNA concentration of MCT8 was lower in fasted than in fed mice with a stronger fasting-induced decrease in PPARα KO mice compared to WT mice (about 57% and 19%, respectively, compared to fed mice of the respective genotype; Figure 5A). In pituitary gland, relative mRNA concentration of MCT8 mRNA was not significantly altered (Figure 5A).

Relative mRNA concentration of MCT10 was influenced by fasting in nearly all tissues tested with fasting-induced increases in liver, hypothalamus and pituitary gland and decreases in kidney, heart and thyroid gland (Figure 5B). In liver, fasting increased MCT10 mRNA concentration in PPARα KO mice about 2.2-fold compared to fed mice of the same genotype whereas no change was seen in WT mice (Figure 5B). MCT10 mRNA level in hypothalamus was slightly higher in fasted compared to fed PPARα KO mice (Figure 5B). Relative MCT10 mRNA concentration in kidney was about 21% lower in fasted compared to fed PPARα KO mice whereas no significant reduction upon fasting was observed in WT mice (Figure 5B). In hearts of WT mice, relative mRNA level of MCT10 was about 29% lower in fasted compared to fed animals, whereas reduction in PPARα KO mice upon fasting was less pronounced (about 18% compared to fed PPARα KO mice; Figure 5B). MCT10 mRNA concentration was lower in thyroid gland of PPARα KO compared to WT mice and was reduced upon fasting about 30% in both genotypes (Figure 5B).

**Figure 6:**
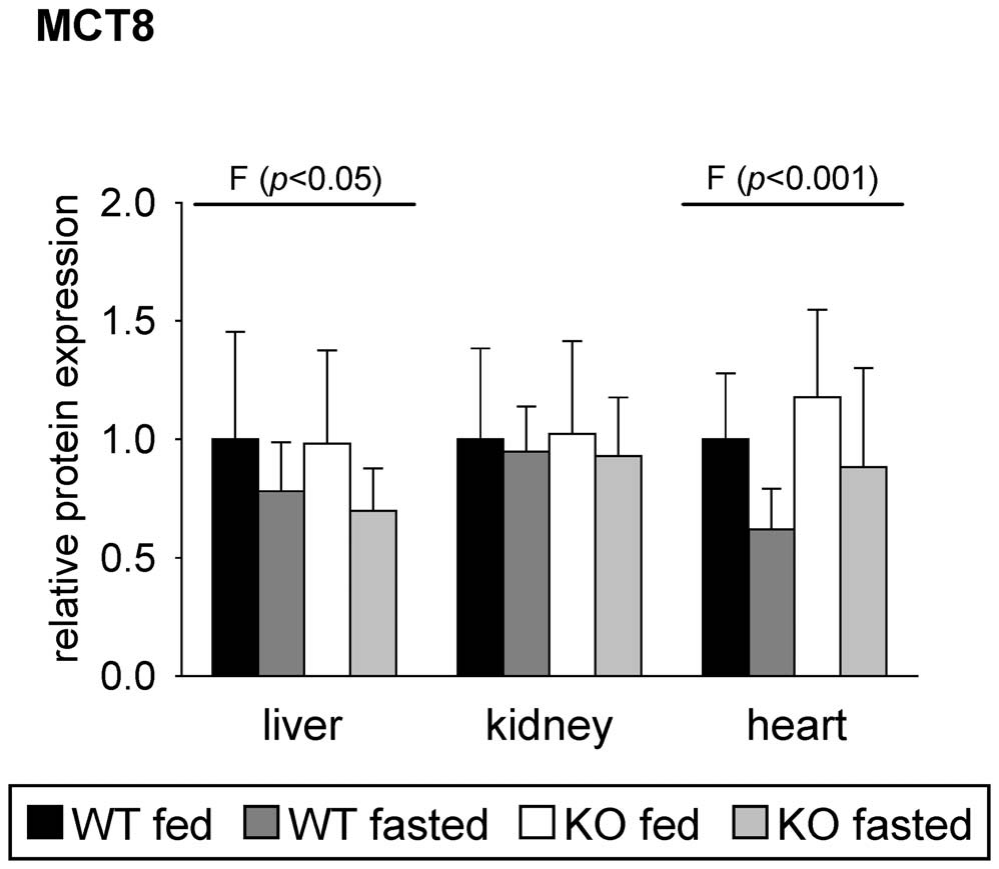
Relative protein expression of monocarboxylate transporter (MCT)-8 in mouse tissues in response to fasting and PPARα. Values represent means ± SD of relative protein expression of wildtype (WT) and PPARα knockout (KO) mice that were fed *ad libitum* or fasted for 48 h (n = 16). Data were analyzed by two-way ANOVA. Classification factors were genotype, fasting, and the interaction between both factors. *P*-values revealed by two-way ANOVA are noted above the figures. Individual means of the treatment groups were compared by Tukey's test in case of variance homogeneity. In case of variance heterogeneity, as revealed by Levene's test, individual means were compared by Games Howell test. No significant differences in post-hoc comparison were revealed.

MCT8 protein expression was analyzed in total tissue lysates of liver, kidney and heart of the mice using western blotting (Figure 6 and Figure S1). MCT8 protein expression was influenced by fasting in liver and heart of mice. In accordance with mRNA analysis, fasting led to a reduction of MCT8 protein expression in liver and heart of mice. Nevertheless, the observed reductions of about 20% in liver and of about 32% in hearts of fasted mice compared to the corresponding fed animals narrowly failed the significance level (Figure 6). No differences in MCT8 protein expression between the four groups of mice were observed in the kidney (Figure 6), which is in contrast to mRNA data (Figure 5B).

## Discussion

Metabolic adaptation is critical to survival of any organism during starvation. In this context, the shuttling of metabolites like ketone bodies and lactate from producing tissues to tissues that use them as energy fuel and for gluconeogenesis is of central importance. These transport processes as well as transport of thyroid hormones and aromatic amino acids are mediated by members of the MCT family. Here, we show that fasting markedly altered the expression of the MCT isoforms 1, −2, −4, −8 and −10 in a tissue-specific manner. Liver and kidney represent crucial tissues in adaptation to energy deprivation by providing ketone bodies as energy fuel and maintaining blood glucose levels by gluconeogenesis from alternative precursors. We were able to show an up-regulation of MCTs that are mainly involved in the hepatic and renal transport of ketone bodies and lactate upon fasting. We assume that the changes of MCT expression are fasting-induced adaptations to maintain the energy supply of tissues. Comparison of WT with PPARα KO mice showed that regulation of the MCT isoforms in fasting conditions is also influenced by PPARα.

In mammals, the main mechanism in adaptation during fasting is the use of fatty acids that are released from adipose tissue as the main energy source and the minimization of glucose utilization. The inability of PPARα KO mice to use these fatty acids as energy fuel due to the lack of induction of β-oxidation and ketogenesis [32], [40], [41] was apparent in higher levels of NEFA in plasma and triacylglycerols in livers of fasted PPARα KO compared to fasted WT mice and also in the lacking increase of the plasma concentration of 3-hydroxybutyrate in PPARα KO mice upon fasting. The impairment of ketogenesis in PPARα KO mice is further demonstrated by the low HMGCS2 mRNA abundance in the liver of fasting PPARα KO mice compared to a strong induction in WT mice upon fasting. In the thyroid gland, the regulation of HMGCS2 mRNA level was completely different with higher levels in the fed state and stronger fasting-induced increase of HMGCS2 mRNA in PPARα KO compared to WT mice. However, the meaning of this unexpected finding remains to be established.

The finding that both genotypes showed a higher expression of MCT1 in liver and kidney upon fasting indicates that neither PPARα nor increased ketone body concentrations are obviously involved in fasting-induced up-regulation of MCT1. We suggest that up-regulation of MCT1 in liver and kidney of fasted mice occurs in the context of its import function for lactate, which is used as gluconeogenetic precursor and may be a regulatory action to compensate the decreasing lactate concentrations in plasma upon fasting [41], [42]. Since mRNA concentrations of MCT1 in livers were not changed, the fasting-induced up-regulation of MCT1 may involve both transcriptional and post-transcriptional mechanisms dependent on fasting endurance. After 24 h of fasting, an up-regulation of MCT1 mRNA could be observed in livers of both rats and pigs [30], [31]. MCT2, which resembles MCT1 in providing lactate for gluconeogenesis, is also up-regulated in the liver of mice upon fasting. However, unlike MCT1, it is down-regulated in kidney upon fasting which may be related with the different physiological roles of both MCT isoforms in kidney. Whereas MCT1 is expressed in proximal tubulus cells that are engaged in gluconeogenesis, MCT2 is found in distal parts of the nephron [43]. In the heart, lactate and glucose are used for oxidation in the fed state, whereas fatty acids are the preferred metabolic fuel in fasted states [44]. Whereas expression of MCT1 was not changed, mRNA concentration of MCT2 and MCT4 decreased upon fasting in hearts of mice of both genotypes which might reflect the decreasing lactate oxidation rate upon fasting. Furthermore, MCT2 mRNA concentration in hearts was higher in PPARα KO than in WT mice. This might be related to higher rates of glycolysis and lactate production that were found in hearts of PPARα KO mice compared to WT mice [45], [46]. However, examination of MCT2 protein expression using total tissue lysates did not reveal any relevant changes in hearts. In colonocytes, MCT1 plays an important role in the absorption of luminal short chain fatty acids [47] and its expression and surface localization is enhanced by substrates involving NFκB and G-protein coupled receptor activation [26], [48]. Though we observed a down-regulation of MCT1 mRNA in small intestine of mice upon fasting in both genotypes, its protein expression was not altered according to western blot analysis. A recent study demonstrated the involvement of MCT4 in butyrate transport in a small intestinal cell line, whereas MCT1 may mediate lactate uptake [49]. MCT4 mRNA concentration was strongly decreased in small intestine upon fasting in both WT and PPARα KO mice in our study. Further analysis is needed to clarify the distinct roles of MCT1 and MCT4 in transport of monocarboxylates in small intestine. In brain, MCT1, -2 and -4 are involved in shuttling of lactate, which is released by astrocytes and taken up by neurons for oxidation, and functions also in memory formation and the control of food intake [10]. According to their selective cellular distribution, MCT1 and MCT4 may be involved predominantly in lactate export, whereas MCT2 preferentially mediates lactate import in neurons [50]. Food deprivation for 48 h has been reported to induce MCT2 mRNA in brainstem of female rats [51]. In our study, MCT1 mRNA concentration in hypothalamus of mice was not affected by 48 h fasting, whereas level of hypothalamic MCT2 mRNA decreased. A recent study suggested that lactate uptake via MCT2 may be involved in control of food intake [52].

Plasma membrane expression and activity of MCT1-4 is dependent on the ancillary proteins CD147 and embigin [1]. For MCT1, the preferred binding partner is CD147 [8], for MCT2 it might be embigin [53]. Analysis of mRNA concentration of both CD147 and embigin in our study showed a tissue-specific regulation that only partly matched the changes observed for expression of MCT1-4.

Besides the metabolic changes in fuel utilization, fasting is also accompanied by a decrease in energy expenditure via down-regulation of the hypothalamus-pituitary-thyroid axis. The thyroid hormone transporters MCT8 and MCT10 show a wide tissue distribution [19], [21], [54], [55]. Here we show reduced levels of MCT8 in response to fasting in liver, kidney, heart, hypothalamus and thyroid gland of mice which might be caused by the reduced levels of thyroid hormones upon fasting. However, other studies showed that hyper- or hypothyroid states did not modulate MCT8 transcript level in hypothalamus [56], [57]. In hypothalamus, MCT8 is notably expressed in regions involved in the negative thyroid hormone feedback on hypothalamic thyreotropin releasing hormone [56] and its down-regulation upon fasting may be involved in this feedback mechanism. Recently, we demonstrated that down-regulation of both TSHβ mRNA expression, and plasma thyroid hormone concentrations upon fasting is stronger in PPARα KO compared to WT mice [58]. In our present study, the concentrations of fT4 in plasma were also lower in PPARα KO mice than in WT mice and a stronger fasting-induced down-regulation in PPARα KO compared to WT mice could be observed regarding the MCT8 mRNA levels in liver and thyroid gland. The physiological relevance of thyroid hormone transport by MCT10 is still unclear [29]. However, the ability of MCT10 to transport aromatic amino acids across plasma membranes is essential for extracellular aromatic amino acid homeostasis control [59]. The liver is the major metabolic organ for catabolism of amino acids and amino acid catabolism is higher in fasted PPARα KO than in WT mice [60]. In our study, we could observe a strong increase of MCT10 mRNA concentration in the liver of PPARα KO mice upon 48 h of fasting compared to fed animals, whereas no significant induction was observed in WT mice. MCT10 is strongly involved in the transport of aromatic amino acids. The increased expression of MCT10 in liver of PPARα KO mice is presumably associated with the stimulated protein catabolism. In kidney, heart and thyroid gland, MCT10 mRNA concentration was decreased upon fasting of mice. Thus, further information on tissue-specific physiological functions of MCT10 is needed to estimate the relevance of these changes.

Our data show that PPARα does not play a key role in the fasting regulation of MCTs. Though we could already show an up-regulation of MCT1 by PPARα activation [30], the present data revealed that PPARα seems not to be involved in induction of MCT1 upon fasting. For other MCT isoforms as MCT2 and MCT10, a minor impact of PPARα on expression was seen. However, the small differences between PPARα KO and WT mice in the present study do not confirm a decisive role of PPARα in fasting adaptation of the analyzed MCT isoforms.

In summary, the current findings show that fasting changes the expression of MCT isoforms in a tissue-specific manner. Since MCTs play a fundamental role in the transport of energy providing metabolites and hormones involved in regulation of energy homeostasis, we assumed that the observed fasting-induced adaptations of MCT expression seem to ensure an adequate energy supply of tissues during the fasting state. Despite the crucial role of PPARα in fasting adaptations, it appears to have only minor impact on regulation of MCTs. The current data illustrate the complex regulation of MCT isoforms and may serve as basis for subsequent studies to elucidate the functional impact of these MCT changes on energy supply of cells and also for drug transport. Due to the prominent role of MCT isoforms 1–4 for the intestinal transport and cellular uptake of statins, β-lactam antibiotics or γ-hydroxybutyrate, the energy state of individuals should be considered in assessing the efficacy of treatments with those drugs.

## Supporting Information

Figure S1 Detection of MCT1, MCT2, MCT8 and Cyclophilin A (CypA) in different tissues of mice by western blot analysis. 20 µg of total lysates of the indicated tissues (L, liver; K, kidney; H, heart; SI, small intestine) were analyzed by western blot using (**A**) anti-MCT1, (**B**) anti-MCT2 and (**C**) anti-MCT8 antibodies. The low molecular weight part of each blot was cropped and analyzed using anti-Cyclophilin A antibody. Predicted molecular masses of the proteins are 53 kDa for both MCT1 and MCT2, 60 kDa for MCT8 and 18 kDa for Cyclophilin A.
(TIFF)

Figure S2 Relative mRNA concentration of (A) CD147 and (B) embigin in mouse tissues in response to fasting and PPARα. Values represent means ± SD of relative mRNA concentrations of wildtype (WT) and PPARα knockout (KO) mice that were fed *ad libitum* or fasted for 48 h (n = 16). Data were analyzed by two-way ANOVA. Classification factors were genotype, fasting, and the interaction between both factors. *P*-values revealed by two-way ANOVA are noted above the figures. Individual means of the treatment groups were compared by Tukey's test in case of variance homogeneity. In case of variance heterogeneity, as revealed by Levene's test, individual means were compared by Games Howell test. Horizontal brackets represent differences between groups in post-hoc comparison (**p<0.001, *p<0.05).
(TIF)
