# Supplementary figures and images for: Tissue-Specific Expression of Monocarboxylate Transporters during Fasting in Mice
Source: PLoS One. 2014 Nov 12;9(11):e112118. doi: 10.1371/journal.pone.0112118 (PMC4229183; doi:10.1371/journal.pone.0112118)

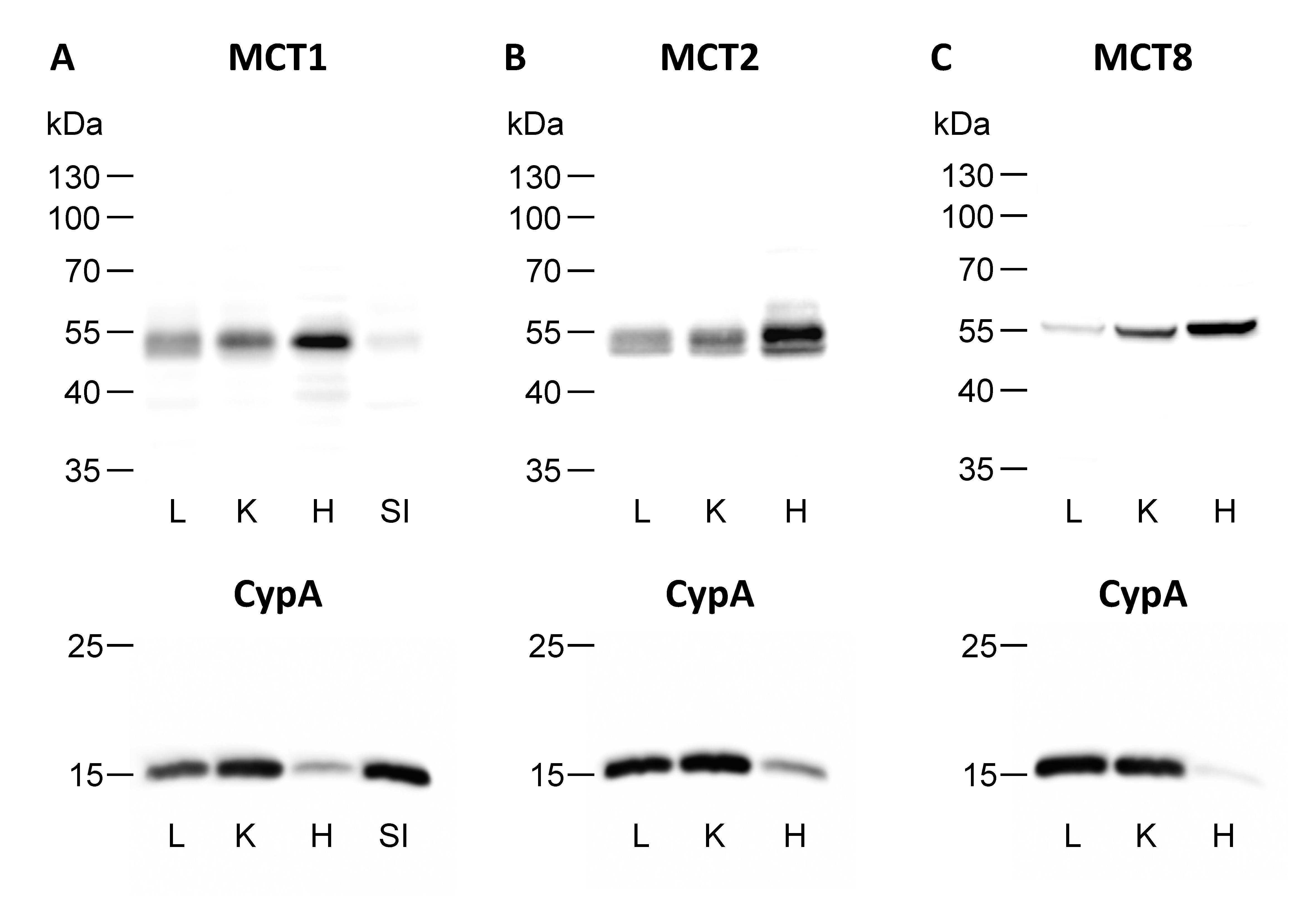

Supplement: Figure S1 — Detection of MCT1, MCT2, MCT8 and Cyclophilin A (CypA) in different tissues of mice by western blot analysis. 20 µg of total lysates of the indicated tissues (L, liver; K, kidney; H, heart; SI, small intestine) were analyzed by western blot using (A) anti-MCT1, (B) anti-MCT2 and (C) anti-MCT8 antibodies. The low molecular weight part of each blot was cropped and analyzed using anti-Cyclophilin A antibody. Predicted molecular masses of the proteins are 53 kDa for both MCT1 and MCT2, 60 kDa for MCT8 and 18 kDa for Cyclophilin A. (TIFF) [file pone.0112118.s001.tiff]

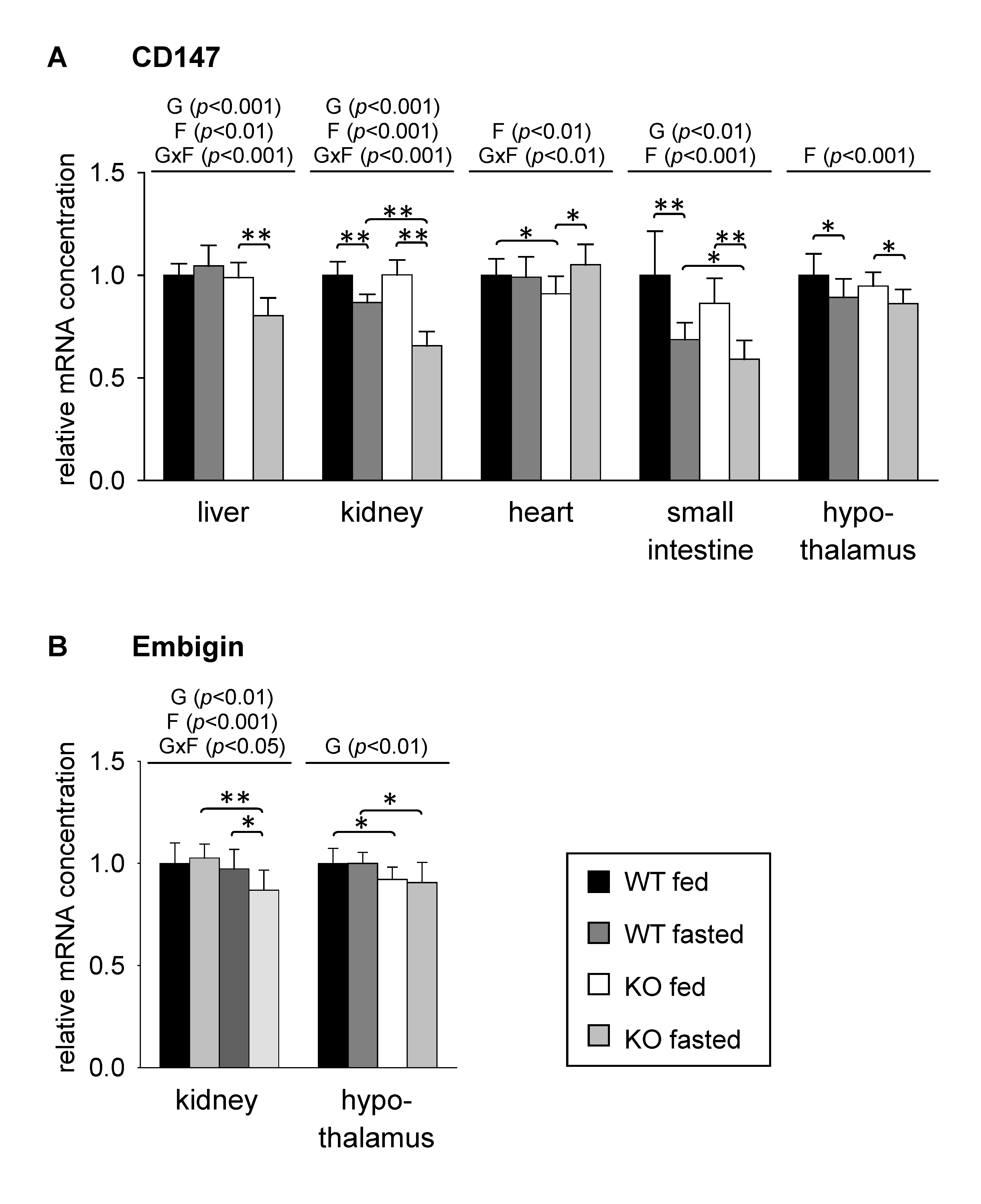

Supplement: Figure S2 — Relative mRNA concentration of (A) CD147 and (B) embigin in mouse tissues in response to fasting and PPARα. Values represent means ± SD of relative mRNA concentrations of wildtype (WT) and PPARα knockout (KO) mice that were fed ad libitum or fasted for 48 h (n = 16). Data were analyzed by two-way ANOVA. Classification factors were genotype, fasting, and the interaction between both factors. P-values revealed by two-way ANOVA are noted above the figures. Individual means of the treatment groups were compared by Tukey's test in case of variance homogeneity. In case of variance heterogeneity, as revealed by Levene's test, individual means were compared by Games Howell test. Horizontal brackets represent differences between groups in post-hoc comparison (**p<0.001, *p<0.05). (TIF) [file pone.0112118.s002.tif]
